# FLIBase: a comprehensive repository of full-length isoforms across human cancers and tissues

**DOI:** 10.1093/nar/gkad745

**Published:** 2023-09-11

**Authors:** Qili Shi, Xinrong Li, Yizhe Liu, Zhiao Chen, Xianghuo He

**Affiliations:** Fudan University Shanghai Cancer Center and Institutes of Biomedical Sciences, Shanghai Medical College, Fudan University, Shanghai 200032, China; Fudan University Shanghai Cancer Center and Institutes of Biomedical Sciences, Shanghai Medical College, Fudan University, Shanghai 200032, China; Fudan University Shanghai Cancer Center and Institutes of Biomedical Sciences, Shanghai Medical College, Fudan University, Shanghai 200032, China; Fudan University Shanghai Cancer Center and Institutes of Biomedical Sciences, Shanghai Medical College, Fudan University, Shanghai 200032, China; Key Laboratory of Breast Cancer in Shanghai, Fudan University Shanghai Cancer Center, Fudan University, Shanghai 200032, China; Shanghai Key Laboratory of Radiation Oncology, Fudan University Shanghai Cancer Center, Fudan University, Shanghai 200032, China; Fudan University Shanghai Cancer Center and Institutes of Biomedical Sciences, Shanghai Medical College, Fudan University, Shanghai 200032, China; Key Laboratory of Breast Cancer in Shanghai, Fudan University Shanghai Cancer Center, Fudan University, Shanghai 200032, China; Shanghai Key Laboratory of Radiation Oncology, Fudan University Shanghai Cancer Center, Fudan University, Shanghai 200032, China

## Abstract

Regulatory processes at the RNA transcript level play a crucial role in generating transcriptome diversity and proteome composition in human cells, impacting both physiological and pathological states. This study introduces FLIBase (www.FLIBase.org), a specialized database that focuses on annotating full-length isoforms using long-read sequencing techniques. We collected and integrated long-read (351 samples) and short-read (12 469 samples) RNA sequencing data from diverse normal and cancerous human tissues and cells. The current version of FLIBase comprises a total of 983 789 full-length spliced isoforms, identified through long-read sequences and verified using short-read exon–exon splice junctions. Of these, 188 248 isoforms have been annotated, while 795 541 isoforms remain unannotated. By overcoming the limitations of short-read RNA sequencing methods, FLIBase provides an accurate and comprehensive representation of full-length transcripts. These comprehensive annotations empower researchers to undertake various downstream analyses and investigations. Importantly, FLIBase exhibits a significant advantage in identifying a substantial number of previously unannotated isoforms and tumor-specific RNA transcripts. These tumor-specific RNA transcripts have the potential to serve as a source of immunogenic recurrent neoantigens. This remarkable discovery holds tremendous promise for advancing the development of tailored RNA-based diagnostic and therapeutic strategies for various types of human cancer.

## Graphical Abstract

**Graphical Abstract:**
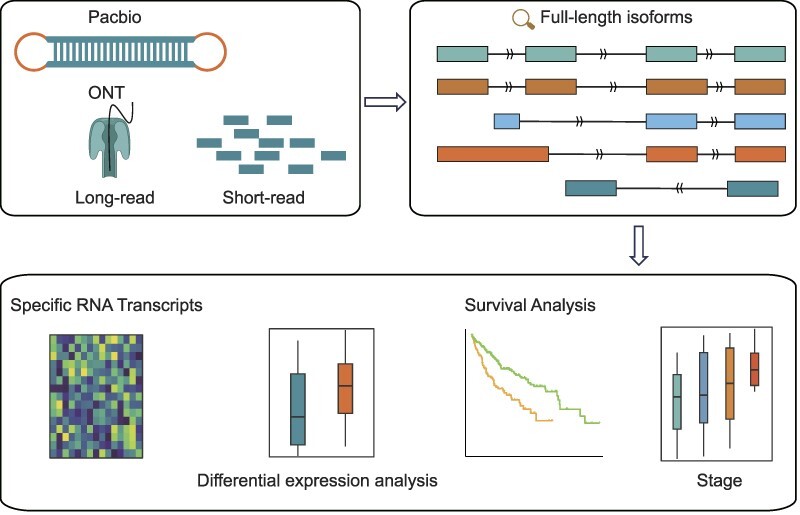

## Introduction

The regulation of transcriptome diversity and proteome composition in human cells involves a range of RNA-level regulatory processes (1,2). These processes, including selectable transcription start sites (TSSs) (3), splicing (4) and polyadenylation (polyA) sites (5), play a crucial role in generating a diverse array of messenger RNA (mRNA) and protein isoforms with distinct structures and functions. Advances in high-throughput RNA sequencing (RNA-seq) and computational methods have greatly facilitated the analysis of transcript changes and the discovery of new transcripts in both normal physiological and pathological states (6,7).

One notable study by Demircioglu *et al.* utilized The Cancer Genome Atlas (TCGA) RNA-seq data to identify a significant number of cancer-associated TSSs and promoters (3). Another study conducted by Hu *et al.* involved reference-based transcript assembly using RNA-seq data obtained from over 1000 cancer cell lines. The findings of this study unveiled numerous transcripts that were previously unidentified (8). Importantly, specific isoform switches have been observed in various diseases, including human cancer. Tumor-preferring transcripts can promote cancer development and progression, making them attractive targets for cancer therapy. For instance, in hepatocellular carcinoma, the short isoform of BIN1 is expressed in normal tissue, while the upregulated long isoform contributes to carcinogenesis (9). Moreover, the identification of tumor-specific RNA transcripts (tumor-SRTs), which are expressed exclusively in tumor tissues, holds great potential for cancer diagnosis and treatment. Zheng *et al.* demonstrated that tumor-specific transcripts could be detected in blood samples from patients with hepatocellular carcinoma, underscoring their potential as diagnostic markers (10). Oncogenic coding and noncoding tumor-SRTs have been identified in numerous types of cancer (11–13). Furthermore, tumor-specific transcripts have been recognized as a source of immunogenic recurrent neoantigens (14).

Given the importance of isoform analysis, several databases have been developed to facilitate the investigation of transcripts and alternative splicing. Notable examples include RJunBase (www.RJunBase.org) (15), TCGASpliceSeq (https://bioinformatics.mdanderson.org/TCGASpliceSeq) (16) and IntroVerse (https://rytenlab.com/browser/app/introverse) (17), which provide information on the alternative splicing sites of annotated and unannotated transcripts. However, these databases do not focus on the full-length characterization of transcripts. Other resources, such as GEPIA2 (http://gepia2.cancer-pku.cn/) (18), SRTdb (http://www.shenglilabs.com/SRTdb/) (19) and TAiC (http://www.shenglilabs.com/TAiC/) (8), offer valuable resources for transcript analysis. Nevertheless, it is crucial to acknowledge the limitations of *de novo* transcript reconstruction methods relying on short-read RNA-seq, as they often encounter challenges leading to incomplete or inaccurate assemblies. In contrast, long-read sequencing (LR-seq) technologies, such as Pacific Biosciences (PacBio) and Oxford Nanopore Technologies (ONT), have the advantage of producing longer reads (>10 kb) (20), which can accurately capture full-length isoforms. This approach eliminates the need for reference-based transcript assembly, ensuring a more comprehensive representation of transcripts. However, there remains a lack of databases that specifically annotate transcripts based on LR-seq.

To bridge this gap, we have developed FLIBase, a web-based database that focuses on the annotation of full-length isoforms using LR-seq techniques. Our database incorporates meticulous curation processes, utilizing long-read RNA-seq data from distinct normal and cancerous human tissues and cells obtained from public databases. Recognizing the limited depth of full-length sequencing data for transcript quantification, we integrated short-read RNA-seq data from human cancer samples and normal tissues sourced from the TCGA and Genotype-Tissue Expression (GTEx) databases, respectively. FLIBase provides extensive information for each transcript, including its complete nucleotide sequence, precise splice site annotations, expression levels, coding potential and predicted open reading frames (ORFs). These comprehensive annotations empower researchers to undertake various downstream analyses and investigations. Notably, FLIBase offers a significant advantage in uncovering a substantial number of previously unannotated isoforms and tumor-SRTs. This remarkable discovery holds tremendous potential for advancing the development of RNA-based diagnostic and therapeutic strategies customized for various tumor types.

## Materials and methods

### LR-seq data collection and processing

LR-seq data from 198 tumor samples (including breast, cervical, colorectal, esophageal, gastric, kidney, liver, lung and ovarian cancer, glioblastoma, leukemia, lymphoma, melanoma, myeloma, neuroendocrine tumor and sarcoma) and 153 normal samples (including adipose tissue, adrenal gland, brain, breast, heart, liver, lung, muscle, ovary, pancreas, testis, fibroblasts, GM12878, H1, H9, HEK293T and WTC11 cells) were collected. The data included PacBio and ONT sequencing data retrieved from various sources, such as GTEx (21), the Genome Sequence Archive (accession number HRA003557) (22–24), ENCODE (25), Sequence Read Archive [accession numbers PRJNA635275 (26), PRJNA664117 (27), PRJNA565724 (28), PRJEB44348 (29), PRJNA515570 (30), PRJNA640456 (31) and PRJNA639366 (31)], the DNA Data Bank of Japan (accession number DRA010214) (32), the Japanese Genotype-phenotype Archive (JGAD000457) (33) and the European Genome Archive (accession number EGAS00001004819) (34). Raw sequencing reads, if available, or other accessible data in FASTQ, BAM, SAM and GTF formats were downloaded.

The raw PacBio sequencing data (BAM files) were processed using the Iso-Seq (version 3.4.0) workflow, which involved the following steps: (i) Subreads of PacBio sequencing data were combined into circular consensus sequence reads with a prediction accuracy threshold of 0.9. (ii) Full-length reads were generated by removing the 5′ and 3′ cDNA primers using Lima with default parameters. Full-length nonchimeric (FLNC) reads were obtained by filtering out artificial concatemer reads and polyA tails. (iii) High-quality transcripts (FASTQ file) were generated by clustering FLNC reads using the isoseq3 cluster. Processed or downloaded FASTQ reads of PacBio and ONT data were then mapped to the human genome (hg38, GENCODE v35) using minimap2 (version 2.17) (35). The mapped ONT sequencing data (SAM files) were processed with the TALON pipeline (version 5.0) (36) to obtain a merged GTF transcriptome. PacBio isoforms were subsequently collapsed using cDNA_Cupcake (version 19.0.0). Full-length transcripts from all samples were merged into a nonredundant transcriptome using gffcompare (version 0.11.2) (37).

### RNA-seq data collection

Junction quantification data from STAR output and 9870 BAM files of RNA-seq data across 33 different human cancer types were retrieved from TCGA under accession number phs000178.v11.p8. Additionally, 2599 BAM files of RNA-seq data across 22 human normal tissue types were downloaded from the GTEx with accession number phs000424.v8.p2.

### Isoform annotation and filtering

Transcripts in the merged GTF file were retained if all splice junctions were detected in at least five samples in the RNA-seq data. Subsequently, isoforms were classified into six groups by comparing them with a reference transcriptome (GENCODE v.35) (38) using SQANTI3 (version 1.6) (39) and gffcompare. A full-splice match (FSM) was defined as an exact match to known isoforms, while incomplete splice match (ISM) transcripts were those contained in the reference with compatible introns. Novel in catalog (NIC) isoforms harbored known splice sites but novel splice junctions, whereas novel not in catalog (NNC) referred to isoforms with at least one splice site not present in GENCODE v.35. Additionally, we identified unannotated genomic regions that included antisense and intergenic transcripts in the LR-seq data. All ISM transcripts, which could be a result of RNA degradation and incomplete reverse transcription, were removed from the whole transcriptome. Cage peaks and polyA sites were annotated using SQANTI3. ORFs in LR-seq transcripts were predicted using GeneMark (version 5.1) (40). Transcripts were predicted to elicit nonsense-mediated decay (NMD) when the stop codon was located >50 nucleotides upstream of the last exon–exon junction. TSSs that overlapped with transposable elements (TEs) within ±100 bp, as determined by the BEDTools suite (version 2.29.2) (41), were considered as TE-derived transcripts. We proceeded with comparable isoform identification and transcript consolidation, adhering to the following criteria: allowing a maximum of three exonic variations, with a defined threshold of no more than four base pairs per exon. The upper limit for permissible dissimilarity stood at 100 for both transcription start and termination sites. Regarding coding transcripts, it was imperative to ascertain the absence of differences in the ORFs. Ultimately, a predilection was manifested for retaining transcripts of extended length. The entire process was facilitated by a dedicated Python code, accessible on Figshare (https://doi.org/10.6084/m9.figshare.23805993.v2).

### RNA-seq data processing

TCGA and GTEx BAM files were converted to FASTQ files using Samtools (version 1.7). Transcript expression, measured in transcripts per kilobase million (TPM), was quantified by mapping RNA-seq reads to long-read isoforms using Salmon (version 1.5.2) (42).

### Specific RNA transcripts

Tumor-SRTs detected in LR-seq are defined as those found exclusively in tumor samples and not in normal samples. Tumor-SRTs identified through RNA-seq are determined based on the following criteria:

- The median expression level of the transcript in tumors is at least 10-fold higher than the maximum expression level observed in all normal tissues (excluding the testis) within the GTEx dataset, as well as adjacent normal tissues within the TCGA dataset.
- The transcript is expressed (TPM > 0.5) in >5% of tumor samples from at least one TCGA cancer type.

The definition of tissue-specific RNA transcripts (tissue-SRTs) follows a methodology similar to a previous study (19). To calculate the specificity score for each transcript, the following formula is used:


\begin{eqnarray*}{S_t} = \log_2( {{N}} ) - \left( { - \mathop \sum \limits_{i = 1}^N ( {{p_{it}} \times \log_2{p_{it}}} )} \right).\end{eqnarray*}

Here, *S_t_* represents the specificity score of transcript *t*, *N* is the total number of tissue types and *p*_*it*_ denotes the expression ratio of transcript *t* in tissue type *i*. Each transcript is assigned one specificity score and *N* expression ratios. The expression ratio of each transcript across all tissue types is calculated as


\begin{eqnarray*}{p_{it}} = \frac{{{x_{it}}}}{{\mathop \sum \nolimits_{i = 1}^N {x_{it}}}},\end{eqnarray*}

where *x*_*it*_ represents the expression value of transcript *t* in tissue type *i*. A transcript is considered tissue specific if the following conditions are met:

- The largest expression ratio is >2 times greater than the second largest expression ratio.
- The specificity score is >1.

### Clinically associated transcripts

The Wilcoxon rank-sum test was employed to assess the statistical differences in expression levels of transcripts between tumor and paired adjacent normal tissues. Multiple testing corrections were performed using the Benjamini–Hochberg method. Transcripts with a false discovery rate (FDR) <0.05 were considered significantly differentially expressed. The statistical difference in overall and progression-free survival between the high-risk and low-risk groups was assessed using a log-rank test. Hazard ratios and *P*-values were calculated using the ‘survdiff’ function implemented in the R package survival (version 3.2-3). The Kruskal–Wallis test was used to determine the statistical differences in expression levels of transcripts among patients with different tumor stages. An FDR < 0.05 was considered significant.

### Visualization of transcript structure and the expression profile

Each gene’s transcripts were graphically presented using Bioconductor’s trackViewer (version 1.24.2) (43), including detailed representation of TEs. Expression profiles of long-read, TCGA and GTEx short-read sequencing data were plotted using the R package ggplot2 (version ≥3.4.0). The optimal cutoff point was calculated using the ‘surv_cutpoint’ function implemented in the R package survminer (version 0.4.9). Kaplan–Meier survival curves were generated using the R package survminer.

## Database content and usage

### Data summary

**Figure 1.**
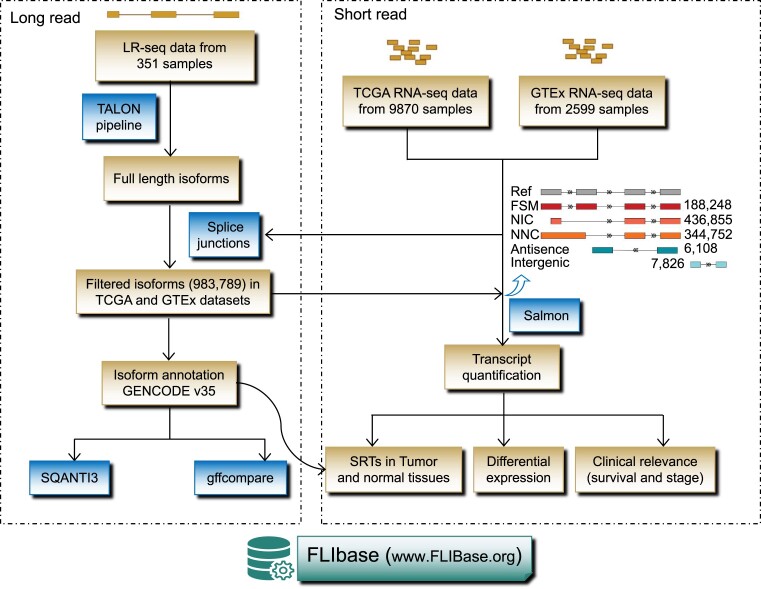
Analytical process for data collection and processing in the FLIBase database. The process includes LR-seq data collection and processing, RNA-seq data collection, isoform annotation and filtering, transcript quantification and downstream analyses.

FLIBase serves as a comprehensive resource by integrating both long-read (351 samples) and short-read (12 469 samples) RNA-seq data from diverse human tissues and cells, encompassing both normal and cancerous samples (Figure 1). The current version of FLIBase comprises a total of 983 789 full-length spliced isoforms, which have been detected using long-read sequences and further validated through short-read exon–exon splice junctions. Among these isoforms, 188 248 are annotated transcripts, while 795 541 are unannotated transcripts. The unannotated transcripts within FLIBase can be classified into four categories: NIC (436 855), NNC (344 752), antisense (6108) and intergenic (7826).

The transcriptome landscape varies across different sample types. For the LR-seq data, the number of identified transcripts in each normal tissue, cell line or cancer type ranges from 5201 in a neuroendocrine tumor sample to 347 049 in 19 stem cell samples (Table 1). Pertinent details concerning sample count and sequencing platform are likewise documented in Table 1. Moreover, in the TCGA dataset, a total of 847 154 transcripts have been identified across 33 distinct cancer types. Notably, the number of expressed transcripts varies among these cancers (Table 2), with the lowest count of 259 510 transcripts observed in lymphoid neoplasm diffuse large B-cell lymphoma (DLBC) and the highest count of 688 614 transcripts in breast invasive carcinoma (BRCA). Similarly, the GTEx dataset encompasses 687 556 transcripts across 22 tissues, with transcript counts ranging from 240 968 in the cervix to 466 119 in the prostate (Table 3). It is worth highlighting that a substantial number of unannotated isoforms are expressed across multiple sample types, consistent with previous research findings (21,28,34). A gene rank indicates the number of detected transcripts across the entire dataset, as well as separately for normal and tumor samples. To address this, we have included relevant information in Supplementary Tables S1–S3. Supplementary Table S4 presents the results of transcripts and tumor-SRTs detected in TCGA samples for each cancer type, while Supplementary Table S5 provides the results of transcripts and tissue-SRTs identified in GTEx samples for each tissue type.

**Table 1.** Distribution of transcripts detected in various samples by LR-seq

Sample	No.	Platform	FSM	NIC	NNC	Antisense	Intergenic	Total
Adipose	1	ONT	12 794	17 052	40	1	0	29 887
Adrenal gland	3	PacBio	29 890	39 811	23 730	375	308	94 114
Brain	24	PacBio and ONT	34 403	64 481	12 422	174	154	111 634
Breast	5	PacBio and ONT	14 966	18 787	874	6	12	34 645
Fibroblast	22	ONT	19 966	40 650	89	1	0	60 706
GM12878	2	PacBio	24 102	39 320	26 769	390	613	91 194
Heart	23	PacBio and ONT	41 898	103 394	46 557	534	554	192 937
Liver	32	PacBio and ONT	29 018	53 737	10 650	165	134	93 704
Lung	8	ONT	20 194	39 395	89	1	0	59 679
Muscle	9	ONT	19 149	36 905	79	1	0	56 134
Ovary	3	PacBio and ONT	32 510	60 206	32 078	396	312	125 502
Pancreas	1	ONT	13 290	19 554	42	0	0	32 886
Stem cell	19	PacBio and ONT	48 053	169 519	126 644	1306	1527	347 049
Testis	1	PacBio	29 606	34 688	34 926	1873	3098	104 191
BreastC	72	PacBio and ONT	47 058	131 885	73 840	506	590	253 879
CRC	24	PacBio and ONT	31 319	46 580	28 082	322	273	106 576
ESC	5	PacBio	13 865	6009	2219	17	37	22 147
GC	10	PacBio	22 138	22 284	11 784	110	186	56 502
KC	1	PacBio	5928	2124	511	13	12	8588
Leukemia	12	PacBio and ONT	33 823	73 888	50 954	651	681	159 997
LiverC	40	PacBio and ONT	31 270	39 644	25 452	422	475	97 263
LungC	29	ONT	23 985	29 069	20 704	423	216	74 397
NET	1	PacBio	3320	1525	337	4	15	5201
OC	3	PacBio	7172	2820	659	11	10	10 672

**Table 2.** Distribution of transcripts expressed in the TCGA RNA-seq datasets

Cancer	FSM	NIC	NNC	Antisense	Intergenic	Total
BRCA	121 633	341 765	221 255	2103	1858	688 614
LUAD	116 083	319 650	195 010	1762	1671	634 176
UCEC	113 936	298 784	194 499	2008	1947	611 174
LUSC	107 209	309 430	184 550	1648	1756	604 593
KIRC	106 648	309 552	180 250	1494	1139	599 083
STAD	103 144	298 533	182 840	1684	1573	587 774
BLCA	103 370	298 212	172 108	1512	1466	576 668
COAD	106 225	292 412	172 756	1356	1271	574 020
LGG	104 814	293 470	163 925	1310	905	564 424
HNSC	98 865	297 382	161 906	1240	1270	560 663
PRAD	100 796	286 916	159 620	1314	939	549 585
KIRP	96 856	278 650	146 804	1198	880	524 388
THCA	92 537	277 651	150 160	1217	785	522 350
CESC	92 042	276 743	148 840	1136	1011	519 772
OV	93 330	270 107	152 518	1716	1523	519 194
SARC	94 324	273 746	135 654	1035	888	505 647
SKCM	89 846	253 216	141 128	1198	1068	486 456
LIHC	87 830	258 741	125 476	1121	1071	474 239
LAML	91 990	249 630	129 761	819	603	472 803
TGCT	85 616	243 274	120 894	959	1187	451 930
PAAD	87 329	246 957	115 034	752	599	450 671
ESCA	77 735	238 270	124 797	1063	1048	442 913
GBM	87 101	236 471	109 379	928	673	434 552
READ	81 377	231 090	111 154	807	767	425 195
PCPG	84 461	233 758	103 884	802	512	423 417
THYM	75 445	216 287	95 242	834	698	388 506
ACC	71 729	203 396	69 898	523	333	345 879
KICH	69 114	190 359	76 959	597	416	337 445
MESO	67 986	193 994	70 356	429	292	333 057
UCS	66 808	194 202	64 403	414	317	326 144
CHOL	60 666	165 307	54 097	318	232	280 620
UVM	59 331	159 600	50 718	317	193	270 159
DLBC	56 349	155 107	47 474	324	256	259 510

**Table 3.** Distribution of transcripts expressed in the GTEx RNA-seq datasets

Tissue	FSM	NIC	NNC	Antisense	Intergenic	Total
Prostate	124 440	268 509	70 829	1085	1256	466 119
Testis	134 339	237 317	68 544	2732	4444	447 376
Small intestine	120 407	261 001	60 898	898	1173	444 377
Spleen	114 724	255 485	64 274	931	1179	436 593
Ovary	115 375	255 653	55 432	819	911	428 190
Liver	107 475	252 032	60 119	709	800	421 135
Lung	111 019	242 424	42 151	566	788	396 948
Skin	109 814	242 280	40 889	516	785	394 284
Breast	109 768	236 185	40 396	623	794	387 766
Colon	108 934	238 103	39 023	506	720	387 286
Esophagus	107 826	237 286	36 926	445	620	383 103
Thyroid	107 678	231 839	38 124	643	744	379 028
Uterus	105 843	229 539	38 386	565	650	374 983
Stomach	105 010	232 560	36 186	435	603	374 794
Brain	108 927	223 247	35 037	546	633	368 390
Heart	101 607	228 025	35 356	499	753	366 240
Adrenal gland	102 978	226 629	33 061	603	679	363 950
Kidney	102 871	224 493	35 098	579	725	363 766
Pancreas	97 438	222 117	33 219	392	475	353 641
Muscle	95 267	213 509	34 371	365	494	344 006
Bladder	76 559	163 244	10 800	130	233	250 966
Cervix	73 965	156 890	9773	138	202	240 968

### Web design and interface

**Figure 2.**
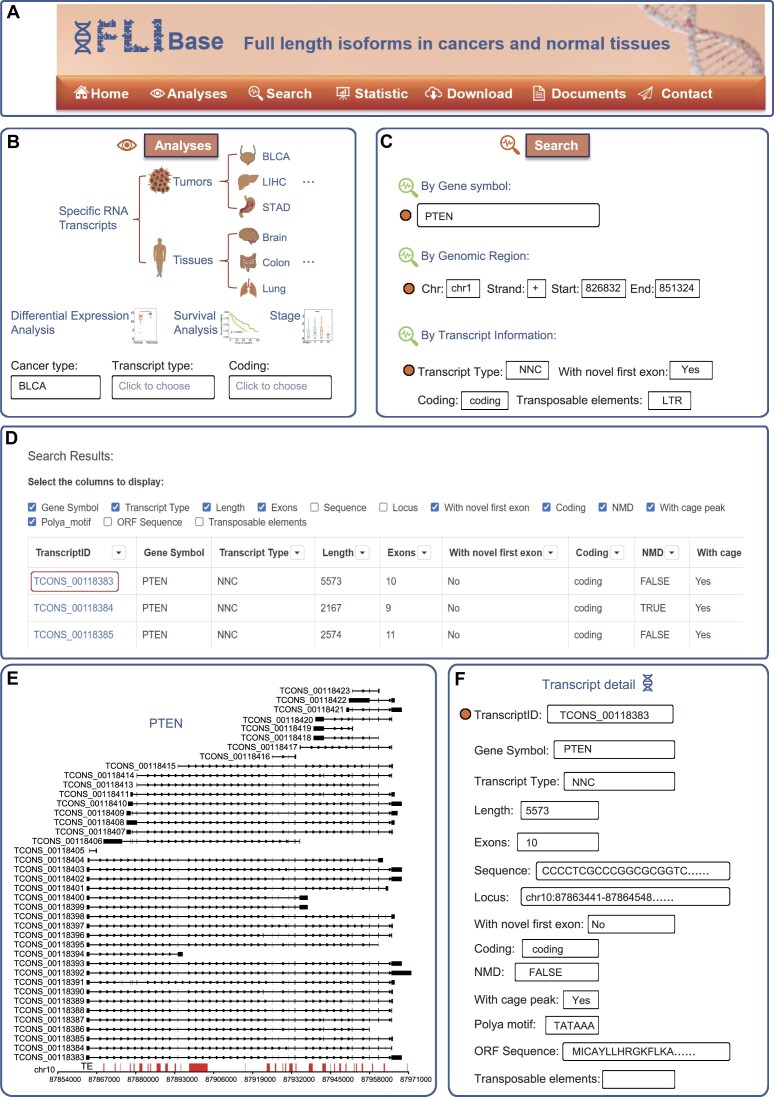
Overview of FLIBase. (**A**) Main user interfaces of the database. (**B**) Four key modules in the ‘Analyses’ section: ‘Specific RNA Transcripts’, ‘Differential Expression Analysis’, ‘Survival Analysis’ and ‘Stage’. (**C**) The ‘Search’ section offers three different search options: gene name, genomic location and transcript information. (**D**) An example of querying a gene symbol on the ‘Search’ page. (**E**) An example of the structure of all transcripts for a specific gene. (**F**) An example of detailed information about a transcript.

FLIBase offers a user-friendly interface and an extensive range of functionalities for analyzing, searching and downloading desired results. Figure 2A showcases the main user interfaces of the database. The ‘Analyses’ page houses four key function modules (Figure 2B): (i) ‘Specific RNA Transcripts’ enables users to query tumor- and tissue-SRTs; (ii) ‘Differential Expression Analysis’ facilitates the investigation of differentially expressed isoforms between tumors and their paired adjacent normal tissues; (iii) ‘Survival Analysis’ allows for the identification of transcripts significantly associated with patients’ overall and progression-free survival within each cancer type; and (iv) ‘Stage’ enables users to explore transcripts significantly associated with tumor stage. For instance, leveraging the TCGA and GTEx RNA-seq datasets, users can extract all transcripts expressed in any cancer type but not in normal tissues.

The ‘Search’ page provides users with three distinct search options: gene name, genomic location and transcript information (Figure 2C). These search methods allow users to retrieve specific transcripts of interest. The genomic location search enables users to specify chromosomes, strands, and genomic start and end positions, facilitating precise transcript retrieval. Additionally, users can utilize specific transcript information to further refine their search results. This includes comparing transcript types to a reference, checking the annotation status of the first exon in a transcript, assessing the coding ability of the transcript and identifying overlaps between TEs and TSSs. By way of illustration, searching for the gene name PTEN enables users to access all detected transcripts associated with PTEN and their relative genomic positions (Figure 2D and E). Furthermore, users can utilize column-based filters within the results field to pinpoint the specific transcript(s) of interest. Once researchers navigate to the details page, FLIBase provides a comprehensive range of information for each transcript (Figure 2F). This includes the transcript ID, associated gene symbol, transcript type in comparison to the reference (FSM, NIC, NNC, antisense and intergenic), transcript length, number of exons, complete nucleotide sequence, genomic location, annotation status of the first exon, coding capacity, prediction of NMD activation, overlap between TSS and CAGE peaks, polyA motif, predicted ORF, and overlap between TEs and TSSs within ±100 bp.

### Applications of FLIBase

FLIBase, with its comprehensive annotation of full-length transcripts, offers valuable insights into the dysregulation of genes and their potential roles in oncogenesis. By utilizing the ‘Specific RNA Transcripts’ function within the ‘Analyses’ section of FLIBase, we investigated the dysregulation of the receptor tyrosine kinase c-MET (MET) and its implications in cancer (44).

Within the FLIBase database, we identified multiple tumor-specific unnamed MET transcripts when filtering the MET genes in the results column. Considering the reported genomic alterations of MET, such as gene amplification, mutations and fusions, these unnamed transcripts provide new avenues for exploring the diverse landscape of MET dysregulation in cancer. One specific transcript, MET-u1 (TCONS_00923684), was selected as an example due to its unannotated TSS region, which overlaps with L1PA2, a LINE-1 subfamily TE. This finding is of interest since TEs have been associated with the activation of oncogenes, suggesting a potential role for MET-u1 in oncogenic processes.

**Figure 3.**
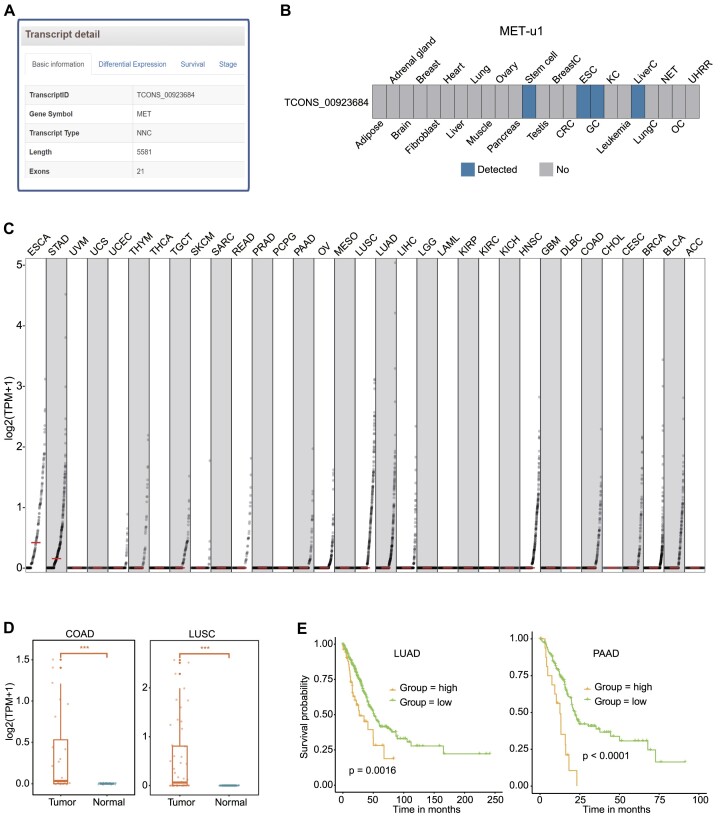
An example of tumor-SRTs. (**A**) Detailed transcript information of MET-u1. (**B**) Detection of MET-u1 in various types of LR-seq data samples. (**C**) Dot plot showing the expression level of MET-u1 in TCGA tumor samples. (**D**) Box plots illustrating the expression of MET-u1 in paired COAD and LUSC samples. (**E**) Kaplan–Meier plots demonstrating overall survival associated with MET-u1 expression in LUAD and PAAD.

MET-u1 is a full-length transcript spanning 5581 bp, classified as an NNC-type transcript, and consisting of 21 exons (Figure 3A). Further details about MET-u1, including its sequence and other relevant information, can be accessed in the FLIBase database. Analysis of the LR-seq dataset revealed the detection of MET-u1 exclusively in esophageal, gastric and liver cancers, while its expression was absent in normal tissues (Figure 3B). Consistent with the LR-seq data, the TCGA RNA-seq dataset demonstrated the expression of MET-u1 across various cancer types, exhibiting notable heterogeneity among patients (Figure 3C). In particular, esophageal and gastric cancers exhibited the highest levels of MET-u1 expression, aligning with the LR-seq findings. Conversely, analysis of normal tissues within the GTEx dataset showed no expression of MET-u1, as depicted in Supplementary Figure S1. Subsequently, specific cancer types were selected to explore the differential expression, survival and stage associations of MET-u1. In the differential expression analysis, we showed that MET-u1 expression was restricted to colon adenocarcinoma (COAD) and lung squamous cell carcinoma (LUSC) tumors, while it was absent in corresponding paracancerous tissues (Figure 3D). This observation indicates potential tumor-specific regulation of MET-u1 expression. Figure 3E demonstrates that high MET-u1 expression is significantly associated with poor prognosis in lung adenocarcinoma (LUAD) and pancreatic adenocarcinoma (PAAD), highlighting the potential prognostic value of MET-u1 in these cancer types.

## Discussion and perspectives

The ENCODE study revealed that a significant portion of the genome (62% of genomic bases) has the potential to be transcribed, indicating the presence of numerous unidentified transcription products (45). However, the prevalent short-read RNA-seq technology poses challenges in fully assembling full-length mRNA isoforms. To address this limitation, we have developed the first comprehensive database of full-length transcripts in human cancer and normal tissues, utilizing LR-seq data. Our database offers a range of analysis, search and download functions, enabling comprehensive exploration and filtering of transcriptomic data. Importantly, we have identified a substantial number of unannotated and tumor-specific transcripts, providing opportunities for investigating their roles in cancer development. For instance, the database allows users to search for an intergenic noncoding transcript derived from an long terminal repeat (LTR), which was previously reported to exert oncogenic activities in promoting hepatocellular carcinoma *in vitro* and *in vivo* (10).

Dysregulation of RNA-related processes can significantly impact the transcriptomic and proteomic landscape of cancer. Emerging evidence suggests that the dysregulation of transcriptional processes associated with tumorigenesis could serve as a broad yet unexplored target for novel immunotherapy approaches. Alternative splicing events, such as intron retention (46), exitron generation (47) and TE-associated splicing (48), contribute to proteome diversity and can generate tumor-specific neoantigens. Long-read RNA-seq technology proves valuable in accurately inferring ORFs and reliably predicting NMD transcripts due to its ability to accurately capture full-length transcripts. In a recent study, aberrant splice isoforms detected through full-length transcriptome sequencing were identified as potential neoantigens in non-small cell lung cancer (32).

As full-length sequencing technology continues to advance, the availability of LR-seq data from human cancerous and normal tissues will increase. FLIBase will be regularly updated to incorporate additional data from diverse tissue types. Furthermore, recent studies have employed LR-seq at the single-cell level (49,50). Thus, we plan to integrate single-cell full-length RNA-seq data into our database to explore isoform heterogeneity across different cell types and states. Through more rigorous investigation and mining of the cancer transcriptome at the transcript level, we anticipate uncovering new insights into cancer diagnostics and therapeutics.

## Supplementary Material

gkad745_supplemental_files

## Data Availability

FLIBase is a freely accessible online database available at www.FLIBase.org. The source code of comparable isoform identification and transcript consolidation is available on Figshare (https://doi.org/10.6084/m9.figshare.23805993.v2).

## References

[1] Wang E.T. , Sandberg R., Luo S., Khrebtukova I., Zhang L., Mayr C., Kingsmore S.F., Schroth G.P., Burge C.B. Alternative isoform regulation in human tissue transcriptomes. Nature. 2008; 456:470–476. 18978772 10.1038/nature07509 PMC2593745

[2] Climente-Gonzalez H. , Porta-Pardo E., Godzik A., Eyras E. The functional impact of alternative splicing in cancer. Cell Rep. 2017; 20:2215–2226. 28854369 10.1016/j.celrep.2017.08.012

[3] Demircioglu D. , Cukuroglu E., Kindermans M., Nandi T., Calabrese C., Fonseca N.A., Kahles A., Lehmann K.V., Stegle O., Brazma A. et al. A pan-cancer transcriptome analysis reveals pervasive regulation through alternative promoters. Cell. 2019; 178:1465–1477. 31491388 10.1016/j.cell.2019.08.018

[4] Cancer Genome Atlas Research Network Kahles A. , Lehmann K.V., Toussaint N.C., Huser M., Stark S.G., Sachsenberg T., Stegle O., Kohlbacher O., Sander C. et al. Comprehensive analysis of alternative splicing across tumors from 8,705 patients. Cancer Cell. 2018; 34:211–224. 30078747 10.1016/j.ccell.2018.07.001 PMC9844097

[5] Zhao Z. , Xu Q., Wei R., Wang W., Ding D., Yang Y., Yao J., Zhang L., Hu Y.Q., Wei G. et al. Cancer-associated dynamics and potential regulators of intronic polyadenylation revealed by IPAFinder using standard RNA-seq data. Genome Res. 2021; 31:2095–2106. 34475268 10.1101/gr.271627.120 PMC8559711

[6] Attig J. , Young G.R., Hosie L., Perkins D., Encheva-Yokoya V., Stoye J.P., Snijders A.P., Ternette N., Kassiotis G. LTR retroelement expansion of the human cancer transcriptome and immunopeptidome revealed by *de novo* transcript assembly. Genome Res. 2019; 29:1578–1590. 31537638 10.1101/gr.248922.119 PMC6771403

[7] Li S. , Hu Z., Zhao Y., Huang S., He X. Transcriptome-wide analysis reveals the landscape of aberrant alternative splicing events in liver cancer. Hepatology. 2019; 69:359–375. 30014619 10.1002/hep.30158

[8] Hu W. , Wu Y., Shi Q., Wu J., Kong D., Wu X., He X., Liu T., Li S. Systematic characterization of cancer transcriptome at transcript resolution. Nat. Commun. 2022; 13:6803. 36357395 10.1038/s41467-022-34568-z PMC9649690

[9] Hu Z. , Dong L., Li S., Li Z., Qiao Y., Li Y., Ding J., Chen Z., Wu Y., Wang Z. et al. Splicing regulator p54^nrb^/non-POU domain-containing octamer-binding protein enhances carcinogenesis through oncogenic isoform switch of MYC box-dependent interacting protein 1 in hepatocellular carcinoma. Hepatology. 2020; 72:548–568. 31815296 10.1002/hep.31062

[10] Zheng Q. , Zhao J., Yu H., Zong H., He X., Zhao Y., Li Y., Wang Y., Bao Y., Li Y. et al. Tumor-specific transcripts are frequently expressed in hepatocellular carcinoma with clinical implication and potential function. Hepatology. 2020; 71:259–274. 31173389 10.1002/hep.30805

[11] Guo W. , Hu Z., Bao Y., Li Y., Li S., Zheng Q., Lyu D., Chen D., Yu T., Li Y. et al. A LIN28B tumor-specific transcript in cancer. Cell Rep. 2018; 22:2016–2025. 29466730 10.1016/j.celrep.2018.02.002

[12] Jang H.S. , Shah N.M., Du A.Y., Dailey Z.Z., Pehrsson E.C., Godoy P.M., Zhang D., Li D., Xing X., Kim S. et al. Transposable elements drive widespread expression of oncogenes in human cancers. Nat. Genet. 2019; 51:611–617. 30926969 10.1038/s41588-019-0373-3 PMC6443099

[13] Wu Y. , Zhao Y., Huan L., Zhao J., Zhou Y., Xu L., Hu Z., Liu Y., Chen Z., Wang L. et al. An LTR retrotransposon-derived long noncoding RNA lncMER52A promotes hepatocellular carcinoma progression by binding p120-catenin. Cancer Res. 2020; 80:976–987. 31874857 10.1158/0008-5472.CAN-19-2115

[14] Shah N.M. , Jang H.J., Liang Y., Maeng J.H., Tzeng S.C., Wu A., Basri N.L., Qu X., Fan C., Li A. et al. Pan-cancer analysis identifies tumor-specific antigens derived from transposable elements. Nat. Genet. 2023; 55:631–639. 36973455 10.1038/s41588-023-01349-3 PMC13152471

[15] Li Q. , Lai H., Li Y., Chen B., Chen S., Li Y., Huang Z., Meng Z., Wang P., Hu Z. et al. RJunBase: a database of RNA splice junctions in human normal and cancerous tissues. Nucleic Acids Res. 2021; 49:D201–D211. 33179749 10.1093/nar/gkaa1056 PMC7779070

[16] Ryan M. , Wong W.C., Brown R., Akbani R., Su X., Broom B., Melott J., Weinstein J. TCGASpliceSeq: a compendium of alternative mRNA splicing in cancer. Nucleic Acids Res. 2016; 44:D1018–D1022. 26602693 10.1093/nar/gkv1288 PMC4702910

[17] Garcia-Ruiz S. , Gustavsson E.K., Zhang D., Reynolds R.H., Chen Z., Fairbrother-Browne A., Gil-Martinez A.L., Botia J.A., Collado-Torres L., Ryten M. IntroVerse: a comprehensive database of introns across human tissues. Nucleic Acids Res. 2023; 51:D167–D178. 36399497 10.1093/nar/gkac1056 PMC9825543

[18] Tang Z. , Kang B., Li C., Chen T., Zhang Z. GEPIA2: an enhanced web server for large-scale expression profiling and interactive analysis. Nucleic Acids Res. 2019; 47:W556–W560. 31114875 10.1093/nar/gkz430 PMC6602440

[19] Shi Q. , Liu T., Hu W., Chen Z., He X., Li S. SRTdb: an omnibus for human tissue and cancer-specific RNA transcripts. Biomark. Res. 2022; 10:27. 35473935 10.1186/s40364-022-00377-1 PMC9044872

[20] Wenger A.M. , Peluso P., Rowell W.J., Chang P.C., Hall R.J., Concepcion G.T., Ebler J., Fungtammasan A., Kolesnikov A., Olson N.D. et al. Accurate circular consensus long-read sequencing improves variant detection and assembly of a human genome. Nat. Biotechnol. 2019; 37:1155–1162. 31406327 10.1038/s41587-019-0217-9 PMC6776680

[21] Glinos D.A. , Garborcauskas G., Hoffman P., Ehsan N., Jiang L., Gokden A., Dai X., Aguet F., Brown K.L., Garimella K. et al. Transcriptome variation in human tissues revealed by long-read sequencing. Nature. 2022; 608:353–359. 35922509 10.1038/s41586-022-05035-y PMC10337767

[22] Chen T. , Chen X., Zhang S., Zhu J., Tang B., Wang A., Dong L., Zhang Z., Yu C., Sun Y. et al. The Genome Sequence Archive family: toward explosive data growth and diverse data types. Genomics Proteomics Bioinformatics. 2021; 19:578–583. 34400360 10.1016/j.gpb.2021.08.001 PMC9039563

[23] CNCB-NGDC Members and Partners Database resources of the National Genomics Data Center, China National Center for Bioinformation in 2023. Nucleic Acids Res. 2023; 51:D18–D28. 36420893 10.1093/nar/gkac1073 PMC9825504

[24] Chen Z. , Shi Q., Zhao Y., Xu M., Liu Y., Li X., Liu L., Sun M., Wu X., Shao Z. et al. Long-read transcriptome landscapes of primary and metastatic liver cancers at transcript resolution. 2023; bioRxiv doi:12 July 2023, preprint: not peer reviewed 10.1101/2023.07.11.548526. PMC10773130 38185659

[25] ENCODE Project Consortium Moore J.E. , Purcaro M.J., Pratt H.E., Epstein C.B., Shoresh N., Adrian J., Kawli T., Davis C.A., Dobin A., Kaul R et al. Expanded encyclopaedias of DNA elements in the human and mouse genomes. Nature. 2020; 583:699–710. 32728249 10.1038/s41586-020-2493-4 PMC7410828

[26] Huang K.K. , Huang J., Wu J.K.L., Lee M., Tay S.T., Kumar V., Ramnarayanan K., Padmanabhan N., Xu C., Tan A.L.K. et al. Long-read transcriptome sequencing reveals abundant promoter diversity in distinct molecular subtypes of gastric cancer. Genome Biol. 2021; 22:44. 33482911 10.1186/s13059-021-02261-x PMC7821541

[27] Leung S.K. , Jeffries A.R., Castanho I., Jordan B.T., Moore K., Davies J.P., Dempster E.L., Bray N.J., O’Neill P., Tseng E. et al. Full-length transcript sequencing of human and mouse cerebral cortex identifies widespread isoform diversity and alternative splicing. Cell Rep. 2021; 37:110022. 34788620 10.1016/j.celrep.2021.110022 PMC8609283

[28] Sun Y.H. , Wang A., Song C., Shankar G., Srivastava R.K., Au K.F., Li X.Z. Single-molecule long-read sequencing reveals a conserved intact long RNA profile in sperm. Nat. Commun. 2021; 12:1361. 33649327 10.1038/s41467-021-21524-6 PMC7921563

[29] Chen Y. , Davidson N.M., Wan Y.K., Patel H., Yao F., Low H.M., Hendra C., Watten L., Sim A., Sawyer C. et al. A systematic benchmark of Nanopore long read RNA sequencing for transcript level analysis in human cell lines. 2021; bioRxiv doi:22April 2021, preprint: not peer reviewed 10.1101/2021.04.21.440736. PMC11978509 40082608

[30] Cheng Y.W. , Chen Y.M., Zhao Q.Q., Zhao X., Wu Y.R., Chen D.Z., Liao L.D., Chen Y., Yang Q., Xu L.Y. et al. Long read single-molecule real-time sequencing elucidates transcriptome-wide heterogeneity and complexity in esophageal squamous cells. Front. Genet. 2019; 10:915. 31636653 10.3389/fgene.2019.00915 PMC6787290

[31] Liu Q. , Hu Y., Stucky A., Fang L., Zhong J.F., Wang K. LongGF: computational algorithm and software tool for fast and accurate detection of gene fusions by long-read transcriptome sequencing. BMC Genomics. 2020; 21:793. 33372596 10.1186/s12864-020-07207-4 PMC7771079

[32] Oka M. , Xu L., Suzuki T., Yoshikawa T., Sakamoto H., Uemura H., Yoshizawa A.C., Suzuki Y., Nakatsura T., Ishihama Y. et al. Aberrant splicing isoforms detected by full-length transcriptome sequencing as transcripts of potential neoantigens in non-small cell lung cancer. Genome Biol. 2021; 22:9. 33397462 10.1186/s13059-020-02240-8 PMC7780684

[33] Namba S. , Ueno T., Kojima S., Kobayashi K., Kawase K., Tanaka Y., Inoue S., Kishigami F., Kawashima S., Maeda N. et al. Transcript-targeted analysis reveals isoform alterations and double-hop fusions in breast cancer. Commun. Biol. 2021; 4:1320. 34811492 10.1038/s42003-021-02833-4 PMC8608905

[34] Veiga D.F.T. , Nesta A., Zhao Y., Deslattes Mays A., Huynh R., Rossi R., Wu T.C., Palucka K., Anczukow O., Beck C.R. et al. A comprehensive long-read isoform analysis platform and sequencing resource for breast cancer. Sci. Adv. 2022; 8:eabg6711. 35044822 10.1126/sciadv.abg6711 PMC8769553

[35] Li H. Minimap2: pairwise alignment for nucleotide sequences. Bioinformatics. 2018; 34:3094–3100. 29750242 10.1093/bioinformatics/bty191 PMC6137996

[36] Wyman D. , Balderrama-Gutierrez G., Reese F., Jiang S., Rahmanian S., Forner S., Matheos D., Zeng W., Williams B., Trout D. et al. A technology-agnostic long-read analysis pipeline for transcriptome discovery and quantification. 2020; bioRxiv doi:18 June 2019, preprint: not peer reviewed 10.1101/672931.

[37] Pertea G. , Pertea M. GFF utilities: GffRead and GffCompare. F1000Research. 2020; 9:ISCB Comm J-304. 10.12688/f1000research.23297.1 PMC7222033 32489650

[38] Frankish A. , Diekhans M., Ferreira A.M., Johnson R., Jungreis I., Loveland J., Mudge J.M., Sisu C., Wright J., Armstrong J. et al. GENCODE reference annotation for the human and mouse genomes. Nucleic Acids Res. 2019; 47:D766–D773. 30357393 10.1093/nar/gky955 PMC6323946

[39] Tardaguila M. , de la Fuente L., Marti C., Pereira C., Pardo-Palacios F.J., Del Risco H., Ferrell M., Mellado M., Macchietto M., Verheggen K. et al. SQANTI: extensive characterization of long-read transcript sequences for quality control in full-length transcriptome identification and quantification. Genome Res. 2018; 28:396–411. 29440222 10.1101/gr.222976.117 PMC5848618

[40] Besemer J. , Borodovsky M. GeneMark: web software for gene finding in prokaryotes, eukaryotes and viruses. Nucleic Acids Res. 2005; 33:W451–W454. 15980510 10.1093/nar/gki487 PMC1160247

[41] Quinlan A.R. , Hall I.M. BEDTools: a flexible suite of utilities for comparing genomic features. Bioinformatics. 2010; 26:841–842. 20110278 10.1093/bioinformatics/btq033 PMC2832824

[42] Patro R. , Duggal G., Love M.I., Irizarry R.A., Kingsford C. Salmon provides fast and bias-aware quantification of transcript expression. Nat. Methods. 2017; 14:417–419. 28263959 10.1038/nmeth.4197 PMC5600148

[43] Ou J. , Zhu L.J. trackViewer: a Bioconductor package for interactive and integrative visualization of multi-omics data. Nat. Methods. 2019; 16:453–454. 31133757 10.1038/s41592-019-0430-y

[44] Guo R. , Luo J., Chang J., Rekhtman N., Arcila M., Drilon A. MET-dependent solid tumours—molecular diagnosis and targeted therapy. Nat. Rev. Clin. Oncol. 2020; 17:569–587. 32514147 10.1038/s41571-020-0377-z PMC7478851

[45] ENCODE Project Consortium An integrated encyclopedia of DNA elements in the human genome. Nature. 2012; 489:57–74. 22955616 10.1038/nature11247 PMC3439153

[46] Smart A.C. , Margolis C.A., Pimentel H., He M.X., Miao D., Adeegbe D., Fugmann T., Wong K.K., Van Allen E.M. Intron retention is a source of neoepitopes in cancer. Nat. Biotechnol. 2018; 36:1056–1058. 30114007 10.1038/nbt.4239 PMC6226333

[47] Wang T.Y. , Liu Q., Ren Y., Alam S.K., Wang L., Zhu Z., Hoeppner L.H., Dehm S.M., Cao Q., Yang R. A pan-cancer transcriptome analysis of exitron splicing identifies novel cancer driver genes and neoepitopes. Mol. Cell. 2021; 81:2246–2260. 33861991 10.1016/j.molcel.2021.03.028 PMC8141048

[48] Merlotti A. , Sadacca B., Arribas Y.A., Ngoma M., Burbage M., Goudot C., Houy A., Rocanin-Arjo A., Lalanne A., Seguin-Givelet A. et al. Noncanonical splicing junctions between exons and transposable elements represent a source of immunogenic recurrent neo-antigens in patients with lung cancer. Sci. Immunol. 2023; 8:eabm6359. 36735774 10.1126/sciimmunol.abm6359

[49] Gupta I. , Collier P.G., Haase B., Mahfouz A., Joglekar A., Floyd T., Koopmans F., Barres B., Smit A.B., Sloan S.A. et al. Single-cell isoform RNA sequencing characterizes isoforms in thousands of cerebellar cells. Nat. Biotechnol. 2018; 36:1197–1202. 10.1038/nbt.4259 30320766

[50] Shi Z.X. , Chen Z.C., Zhong J.Y., Hu K.H., Zheng Y.F., Chen Y., Xie S.Q., Bo X.C., Luo F., Tang C. et al. High-throughput and high-accuracy single-cell RNA isoform analysis using PacBio circular consensus sequencing. Nat. Commun. 2023; 14:2631. 37149708 10.1038/s41467-023-38324-9 PMC10164132

