# Supplementary material for: FLIBase: a comprehensive repository of full-length isoforms across human cancers and tissues
Source: Nucleic Acids Res. 2023 Sep 11;52(D1):D124–33. doi: 10.1093/nar/gkad745 (PMC10767943; doi:10.1093/nar/gkad745)
Supplement: gkad745_supplemental_files [file gkad745_supplemental_files.zip › Supporting Info_FLIBase.pdf]

Supporting Information

FLIBase: a comprehensive repository of full-length isoforms across human cancers and tissues

Qili Shi[1], Xinrong Li[1], Yizhe Liu[1], Zhiao Chen[1,2,3], Xianghuo He[1,2,3,*]

[1]Fudan University Shanghai Cancer Center and Institutes of Biomedical Sciences, Shanghai Medical College, Fudan University, Shanghai 200032, China

[2]Key Laboratory of Breast Cancer in Shanghai, Fudan University Shanghai Cancer Center, Fudan University, Shanghai 200032, China.

[3]Shanghai Key Laboratory of Radiation Oncology, Fudan University Shanghai Cancer Center, Fudan University, Shanghai, 200032, China

* To whom correspondence should be addressed. Tel: 86-21-34777329; Fax: 6-21-64172585; Email: xhhe@fudan.edu.cn

Supplementary Table S1. A gene rank illustrating the number of detected transcripts in the LR-seq datasets.

Supplementary Table S2. A gene rank illustrating the number of detected transcripts in the TCGA RNA-seq datasets

Supplementary Table S3. A gene rank illustrating the number of detected transcripts in the GTEx RNA-seq datasets

Supplementary Table S4. Distribution of tumor-specific RNA transcripts expressed in the TCGA RNA-seq datasets

Cancers	FSM	NIC	NNC	Antisense	Intergenic	Total
ACC	151	798	1259	14	19	2241
BLCA	324	1161	3190	91	138	4904
BRCA	239	1071	2951	76	57	4394
CESC	204	1127	2438	45	56	3870
CHOL	78	612	1318	11	11	2030
COAD	212	1120	2490	56	96	3974
ESCA	578	3344	4963	139	176	9200
GBM	380	1270	2255	46	33	3984
HNSC	337	1255	2478	102	134	4306
KICH	105	349	533	14	9	1010
KIRC	165	431	1408	42	39	2085
KIRP	111	439	1279	26	18	1873
LGG	755	1857	3542	74	38	6266
LIHC	221	460	1348	52	86	2167
LUAD	321	789	2365	92	106	3673
LUSC	497	1586	3534	152	221	5990
MESO	84	346	756	14	20	1220
OV	576	2104	4088	160	157	7085
PAAD	103	379	1018	21	14	1535
PCPG	316	760	1578	17	22	2693
PRAD	84	293	1105	22	25	1529
READ	199	860	1494	49	84	2686

SARC	232	1070	1966	45	43	3356
SKCM	555	987	2428	74	85	4129
STAD	894	3647	6274	145	141	11101
THCA	67	213	666	14	12	972
THYM	362	875	2149	74	81	3541
UCEC	429	1951	4798	75	114	7367
UCS	286	1453	1919	24	38	3720
UVM	188	239	622	10	9	1068

Supplementary Table S5. Distribution of tissue-specific RNA transcripts expressed in the GTEx RNA-seq datasets

Tissues	FSM	NIC	NNC	Antisense	Intergenic	Total
Adrenal gland	694	1141	596	68	62	2561
Bladder	239	510	63	2	3	817
Brain	3326	2565	1195	51	31	7168
Breast	276	547	333	14	23	1193
Cervix	357	891	88	3	3	1342
Colon	91	106	106	2	7	312
Esophagus	62	59	76	3	4	204
Heart	459	832	616	48	84	2039
Kidney	813	520	407	27	36	1803
Liver	1546	2411	2034	98	101	6190
Lung	697	1101	537	21	28	2384
Muscle	1713	3048	1478	28	28	6295
Ovary	786	1580	1474	62	58	3960
Pancreas	488	619	274	18	20	1419
Prostate	766	1002	1309	50	61	3188
Skin	1285	1572	635	32	44	3568
Small intestine	595	772	733	26	33	2159
Spleen	2772	3894	2504	103	152	9425
Stomach	200	189	110	11	5	515
Testis	22010	15842	21483	1938	3111	64384
Thyroid	1059	1537	836	39	49	3520
Uterus	420	506	353	4	14	1297

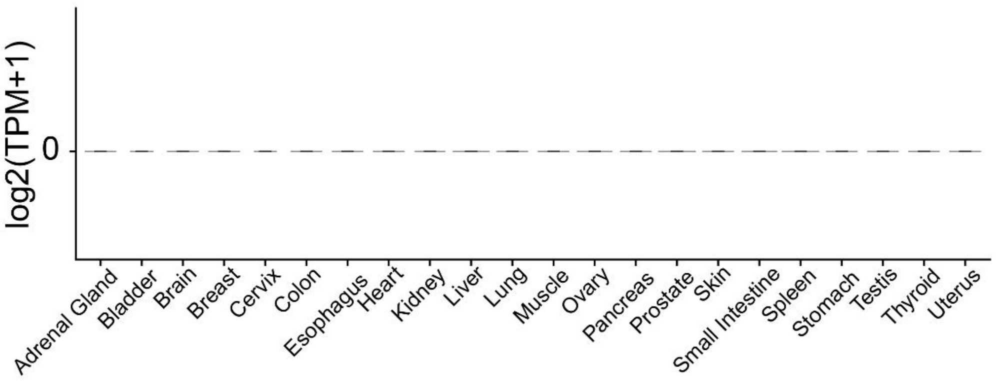

Supplementary Figure S1. Box plot displaying the expression of MET-u1 in the GTEx tissues.
